# Benefits of thermal and distance-filtered imaging for wayfinding with prosthetic vision

**DOI:** 10.1038/s41598-024-51798-x

**Published:** 2024-01-15

**Authors:** Roksana Sadeghi, Arathy Kartha, Michael P. Barry, Paul Gibson, Avi Caspi, Arup Roy, Duane R. Geruschat, Gislin Dagnelie

**Affiliations:** 1 Department of Biomedical Engineering, Johns Hopkins School of Medicine, Baltimore, MD USA; 2 Herbert Wertheim School of Optometry and Vision Science, University of California, Berkeley, CA USA; 3 Department of Biological and Vision Sciences, State University of New York College of Optometry, New York, NY USA; 4 Department of Ophthalmology, Johns Hopkins School of Medicine, Baltimore, MD USA; 5 Pritzker Institute for Biomedical Science and Engineering, Illinois Institute of Technology, Chicago, IL USA; 6 Advanced Medical Electronics Corporation, Maple Grove, MN USA; 7 Jerusalem College of Technology, Jerusalem, Israel; 8 Zikron, Inc., Campbell, CA USA

**Keywords:** Neuroscience, Visual system, Retina, Biomedical engineering, Translational research

## Abstract

Visual prostheses such as the Argus II provide partial vision for individuals with limited or no light perception. However, their effectiveness in daily life situations is limited by scene complexity and variability. We investigated whether additional image processing techniques could improve mobility performance in everyday indoor environments. A mobile system connected to the Argus II provided thermal or distance-filtered video stimulation. Four participants used the thermal camera to locate a person and the distance filter to navigate a hallway with obstacles. The thermal camera allowed for finding a target person in 99% of trials, while unfiltered video led to confusion with other objects and a success rate of only 55% ($p<0.01$). Similarly, the distance filter enabled participants to detect and avoid 88% of obstacles by removing background clutter, whereas unfiltered video resulted in a detection rate of only 10% ($p<0.01$). For any given elapsed time, the success rate with filtered video was higher than with unfiltered video. After 90 s, participants’ success rate reached above 50% with filtered video and 24% and 3% with normal camera in the first and second tasks, respectively. Despite individual variations, all participants showed significant improvement when using the thermal and distance filters compared to unfiltered video. Adding a thermal and distance filter to a visual prosthesis system can enhance the performance of mobility activities by removing clutter in the background, showing people and warm objects with the thermal camera, or nearby obstacles with the distance filter.

## Introduction

Visual perception elicited by electrical stimulation of the neurons in the visual pathway can help to improve functional vision (the ability to perform visual tasks) for people with (near-)total vision loss^1^. With the recent cutting-edge technologies in the biomedical engineering field, there are more than 40 research teams working on different visual neuroprostheses worldwide, three of which have received regulatory approval by the FDA and/or CE mark for commercial use in the United States and Europe: Argus II by Second Sight Medical Products (now Vivani Medical, Inc.), United States; Alpha AMS by Retina Implant AG, Germany; and IRIS by Pixium Vision, France. All three are retinal neuro-prostheses for patients with end-stage retinal degenerative diseases such as retinitis pigmentosa and aged related macular degeneration. However, the manufacturing of these devices is suspended^2–4^.

The most widely used visual prosthesis, with more than 350 users worldwide, is the Argus II epiretinal implant^1^. The Argus II system consists of an implanted device with 60 electrodes that stimulate the inner retinal surface, partially restoring vision in about 22 degrees of the visual field; the external components of the system include a head-mounted camera and a belt pack computer called video processing unit (VPU). The head-mounted camera frames are fed into the VPU, which converts the frame image to grayscale, down-samples it to 60 pixels, uses the image intensity to assign each electrode stimulation parameters, and transfers this information along with power, wirelessly, to the implanted device via a telemetry link in approximately real-time^5^.

Argus II users describe perception with the Argus II as moving shadows in a narrow visual field, and score an average of 2.5 logMAR (approximately 20/6000) with grating visual acuity tests. Functionally, Argus II users fall comfortably within the category of ultra-low vision (ULV), described as “vision so limited that it prevents the individual from distinguishing shape and detail in ordinary viewing conditions” by Jeter et al.^6–8^ Studies of Orientation and Mobility (O&M) in ULV have shown that even a limited amount of residual vision can help with wayfinding by following the lights from the ceiling, windows, and doorways^9,10^. However, ULV may not be enough for obstacle detection and navigation of elevation changes (for example, detecting stairs). People with ULV accomplish O&M activities in daily life using non-visual aids such as a long cane, a guide dog, or a sighted person’s support^3,11^. Using a long cane is arguably the most common non-visual aid for mobility and wayfinding. However, a long cane fails to detect obstacles that are above the ground, and it detects only objects within the cane's reach.

In the totally blind, vision restoration devices such as the Argus II retinal implant have been shown to improve the ability to perform visual tasks in a range of activities such as object detection, direction of motion, and obstacle avoidance, but mainly in high contrast laboratory settings^5,12–15^. However, in real-life scenarios, the contrast and number of objects in the visual field are different from laboratory testing environments, and the visual scene usually contains multiple objects at various distances with different contrasts. Due to the lack of form vision with Argus II, the main information about the objects in the scene is the size of the percepts. Users would generally not be able to determine why a percept is larger, whether it is because there are more objects, or an object is bigger, brighter, or closer. Therefore, as with natural ULV, Argus II users depend on their other senses and use non-visual aids such as a long cane, a guide dog, or a sighted person’s support for O&M activities in daily life.

To compensate for the limited vision with Argus II, image-processing techniques can be used to simplify the image and isolate the desired objects in the field of view to enhance the user's visual ability^16^. For example, a heat-sensitive camera can detect people and warm objects in the room and not show cold objects in the scene. This would be helpful when interacting with people, when walking in a public space looking for a person to talk to, or to avoid bumping into people^17–20^. Similarly, a disparity-based distance filtering system can be used to detect object distances, showing only the objects within an adjustable range, e.g., beyond the reach of a long cane, and present those at an enhanced contrast setting, while removing objects outside of the selected range^21^.

Recently four published studies suggested the benefit of a heat-sensitive camera and disparity-based distance filtering system for Argus II users^18–21^. Three of the studies tested their system in various stationary tasks where the participant had to perform the task while seated^19–21^. He et al., 2020 performed a mobility experiment with four Argus II participants in which a thermal camera was used to improve performance of detecting and either avoiding or approaching people at distances of less than 3m, while using a long cane^18^. However, performance improvements over longer distances, as one might encounter in hospitals or supermarkets, as well as effects on success rates for mobility tasks within time constraints, have been missing in the previous studies, which we addressed here. Also, to isolate the benefit of visual performance with a thermal camera in our study, we asked our participants not to use other aids, including a long cane. Removing mobility aids allowed us to better examine the benefit of filtered prosthetic vision for approaching or avoiding targets with implantees who do not normally use such aids in daily life.

In this study, we used a custom-designed mobile device with three modes of operation: unfiltered video, similar to the standard Argus II camera view, thermal camera view, and disparity-based distance filtered view. The device was connected to the Argus II VPU. We investigated whether use of the thermal or distance filtering could improve success rates at any given response time for two mobility tasks, constrained to 7 min or less. The thermal camera was used to find, walk towards, and shake hands with a person in a quiet hallway. With disparity-based distance filtering, we investigated the same benefits in an object avoidance task while walking through a hallway: Removing the background and showing nearby objects as a bright flash of light can be of great help for obstacle avoidance while walking around.

## Methods

### Custom-designed mobile system

**Figure 1:**
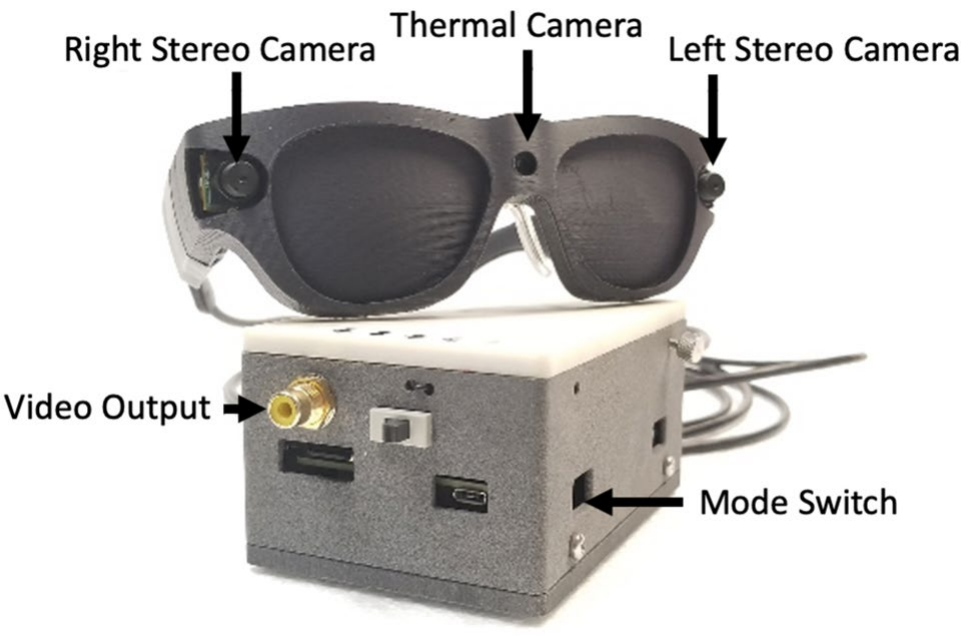
The mobile device with a thermal camera and distance filtering system. The video output of this device is fed to the Argus II system to provide real-time stimulation based on the thermal image or the detected obstacles within the selected range.

To create the mobile system, we used a custom 3D printed spectacle frame with a miniature heat-sensitive infrared camera (Lepton; FLIR Corp.) fitted on the nose bridge. The camera pair for disparity-based stereo used ON Semiconductor Python480 sensors (with a pixel array of 800 × 600 pixels), located on the sides of the frame (Fig. 1). The images from these cameras were fed into an electronics box that generated the scene's thermal- or distance-filtered image. A Nerian processor (Nerian-Vision GmbH) is used for disparity-based image filtering based on the distances of the objects in the field of view. The system can be configured such that only objects within an adjustable distance (low pass), beyond an adjustable distance (high pass), or between two adjustable distances (band pass) are rendered and presented to the Argus II user. There is a button on the side of the processor box that switches between unfiltered (similar to standard Argus II input), thermal-, and distance-filtered imagery. Unfiltered video was obtained by only reading from the left camera of the stereo camera pair. Similar to use with the Argus II regular camera, the unfiltered image was cropped to match the predicted visual field of the array on the retina (approximately 20 degrees diagonally) and downsampled to match the configuration of the implanted electrodes (10 × 6 pixels). Frame rates for both the Argus II camera and the cameras used in these experiments far exceeded the stimulation rates used for the Argus II device, typically six frames/s; the reduction to this stimulation rate was performed by the Argus II VPU.

Based on the processed image, the Argus II Video Processing Unit (VPU) assigns stimulation parameters for each electrode and sends this information to the implant wirelessly through a transmitter coil. This results in real-time stimulation of the electrodes according to image brightness. In the case of filtered video, either the thermal image or the disparity map of the visual scene will trigger stimulation that will be perceived by the user at a target object's location. A detailed description of these two imaging modes has been presented elsewhere^20–22^.

### Study design

We designed and conducted two mobility experiments for Argus II users to perform with either thermal-or distance-filtering and unfiltered video, similar to that of the regular Argus II camera. The study was approved by the Johns Hopkins Medical Institutions IRB and followed the tenets of the Declaration of Helsinki. All subjects signed an informed consent form to participate after being informed of the goals and methods of the study.

#### Participants

Four Argus II users (three male and one female) with end-stage retinitis pigmentosa participated in this study (Table 1). Three participants had no light perception without the Argus II system, and S3 had only limited light perception at the time of testing and could tell whether the room light was on or off. At the time of the experiment, each had been using their Argus II system actively on a daily basis for more than four years. Out of 60 implanted electrodes, each had more than 55 usable active electrodes. S3 and S4 traveled independently from other states to participate and are highly skilled long cane users. S1 and S2 had previously received training using a long cane but now are dependent on a sighted person for navigation and mobility in unfamiliar places.

**Table 1:** Participants’ information.

Subject	Age	Using white cane	Inherent light perception	Years of using Argus II	Number of active Argus II electrodes
S1	87	No	No	12	55
S2	80	No	No	10	56
S3	67	Yes	Bare light perception in non-implanted eye	5	57
S4	58	Yes	No	4	57

#### Experiment 1—Thermal Filter—Locating and approaching a person

**Figure 2:**
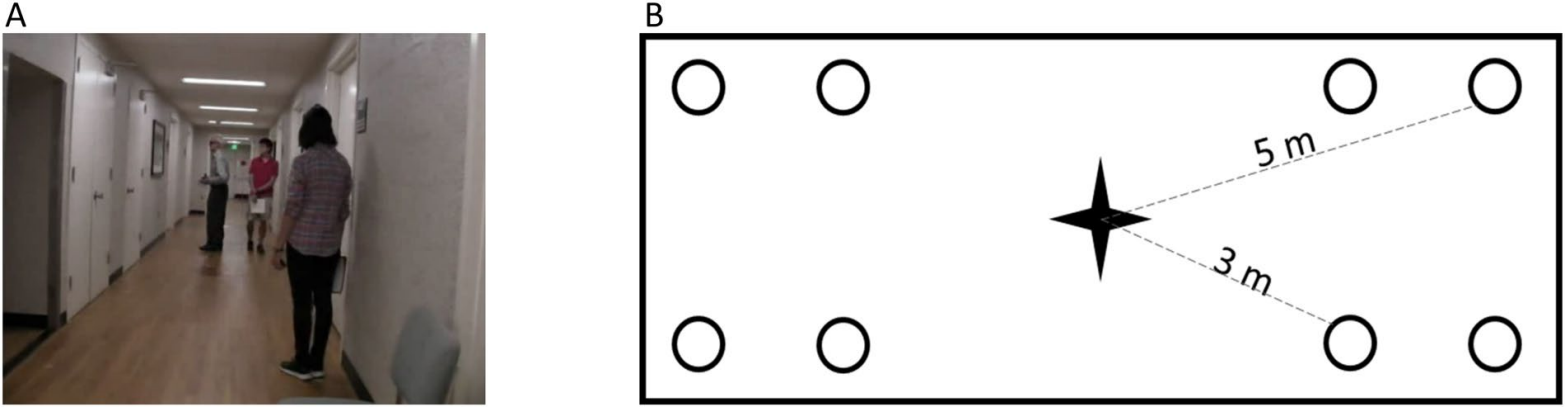
(**A**) An example trial image for experiment 1 when the target was standing 5m away from the starting point and an experimenter was walking with the participant to ensure safety. With the thermal camera, only a person in the camera view will be shown as a bright flash of light. With the normal unfiltered video, however, the white doors, paintings on the wall, ceiling lights and reflections of the ceiling lights on the floor will create flashes as well, which makes it hard to distinguish the flashes for the person from the other flashes. (**B**) In this experiment, the participant started in the middle of the hallway (marked as a filled star) at random orientation and was asked to locate and approach the target person standing 3m or 5m away at one of the eight possible locations (marked as circles).

The participant was guided by one of the experimenters to the middle of an empty rectangular space that was part of a hallway. The starting orientation of the participant was randomized relative to the direction of a silent target person standing 3m or 5m away in one of eight possible locations (Fig. 2). The distances were chosen not to be so close that a long cane could detect them, yet not too far, so the test could be easily repeated for multiple trials without inducing fatigue.

In each trial, the participant was instructed to locate, approach, and shake hands with the target person, all without the aid of a long cane. Two experimenters recorded the accuracy and completion time. To ensure participant safety, one experimenter closely followed the participant without physical contact while the participant moved around independently. The experimenters provided no information about the target person's location at any time. The trial was marked as a success if the participant found and shook hands with the target person without exceeding the 5m boundaries or the 7 min time limit. The 5m boundary was imposed to prevent the participant from walking a long distance in the wrong direction. The 7-min time limit was imposed to avoid frustration for the participant.

After the trial finished, visual stimulation was turned off; the second experimenter distracted and guided the participant back to the starting point, facing a random direction, and gave the starting cue for the next trial. Meanwhile, the target person had moved quietly, without making audible footsteps, to the next trial’s location. It is important to note that while efforts were made to minimize potential confounding factors, including the possibility of participants hearing the target person's movement, we acknowledge that this could play a role; however, noise from the air circulation system and the type of flooring in the hallway reduced the likelihood that the subjects could use such auditory cues. Moreover, the experimenters took precautions to ensure the target person's movements were as unnoticeable as possible.

A practice session was conducted before formal testing trials to familiarize participants with the task. The practice trials included four trials, two trials where the target person was standing at close and two at far locations. In the first session, the performance of the thermal video and the task were explained to the participants. As a practice, participants repeated the task with a thermal camera and with no filtered video, completing four trials for each condition. The practice for the subsequent sessions included four trials only using the condition being tested in that session.

The experiment was repeated for 24 trials with the thermal camera and 24 trials with the regular camera. The 24 trials were randomly ordered with three repetitions of the eight possible target locations. In each test session, one condition was used; the choice was randomized between participants. The experimenters ensured the trials with a thermal camera were performed the same way as with an unfiltered video. Between trials, the participants had a chance to sit down and take a break for as long as they wished. The experiment with each mode was completed within a session lasting up to 4 h.

#### Experiment 2—Distance Filter—Obstacle avoidance in a hallway

**Figure 3:**
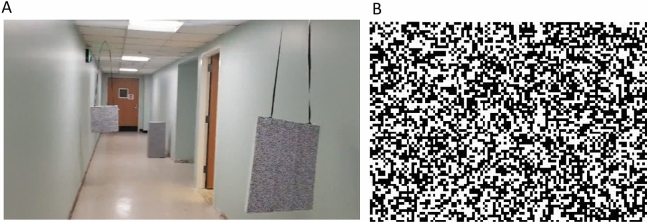
(**A**) Sample setup for the second experiment. In this task, the participant started from one end of a hallway and was asked to walk to the other end without hitting three obstacles. In each trial, the obstacles were placed randomly either on the right or left side of the hallway, and in random order. In this example, the first and second obstacles were hanging from the ceiling, on the right and left side, respectively, and the third obstacle was placed on the floor toward the right side. The distance filter only showed the obstacles within 2m. However, all three obstacles were visible with the unfiltered video. With unfiltered video, the ceiling lights, the reflection of the ceiling lights on the walls and floors, and the doorway might look as similar flashes of light as the obstacles. (**B**) The random black and white pattern covering the obstacles in the second experiment.

The participants were asked to walk 10m in a hallway and locate and avoid three obstacles placed randomly either near the left or right wall at 3 m intervals (Fig. 3a). The 3m distance was selected to be well outside the detection range of a long cane. The obstacles included an empty large cardboard box on the floor and two different-sized Styrofoam boards hanging from the ceiling, featuring printed black and white random dot patterns to facilitate disparity measurements (Fig. 3b).

During the trial, one experimenter closely followed the participant from behind to ensure safety without physical contact while the participant was moving around independently without a long cane. The experimenter provided no information about the location of the objects.

Participants verbally reported (“to the left” or “to the right”) and pointed to upcoming obstacles, with the other experimenter monitoring their camera view on a display to confirm responses accurately corresponded to the objects in view. The experimenter's camera view allowed the experimenter to see in real time whether any retinal stimulation was provided and, if so, precisely what visual stimuli triggered electrode activation. The identity of any stimulus triggering electrode activation, or lack thereof, just prior to the participant’s report of detecting an obstacle was the primary determinant of whether the participant’s response was correct. Participants were not required to report a response every 3m and might have missed obstacles. Additionally, if participants misjudged the size of an obstacle, they might hit it, even if the obstacle was detected. The experimenter recorded the completion time after walking 10m, the number of obstacles that were correctly reported, and the number of obstacles that were hit. The trial was marked a success if all three obstacles were detected and avoided.

When the trial was completed, the participant’s visual stimulation was turned off, and they were guided to the next starting point. Meanwhile, the experimenter moved the obstacles as quietly as possible while the second experimenter occupied the participant. When the setup was ready, the experimenter turned on the participant’s visual stimulation and started the next trial.

The distance filter was set to the low pass mode to show obstacles within the experimenter-set distance of two meters while hiding other objects in the camera view at longer distances. There were 14 trials with the distance filter and 14 trials with unfiltered video. The experimenters ensured the trials with distance filtering were executed the same way as with unfiltered video.

In the first session, the view with a distance filter was explained and demonstrated by performing the task for three trials with a distance filter and three trials with no filter. In other sessions, before the test trials, the participant practiced the task in three trials. In each test session, the task was performed with one mode. The order of tested modes was randomized between subjects.

The participants had the option to sit down at any time during the experiment and take a break as often as they wished. The experiment with each condition was completed within a session lasting up to 4 h.

### Data analysis

We conducted data analysis for both experiments by calculating the cumulative probability of correct performance over time, utilizing both accuracy and completion time. With a time bin size of 10 s, we applied the Kolmogorov–Smirnov test (with a 95% significance level) to compare performance between the thermal or distance filter conditions and unfiltered video.

The plateau in the cumulative probability of success curve was identified if the slope of the cumulative probability of the correct curve was smaller than 0.1 for three consecutive data points. The identified plateau indicates that, after a certain duration, more effort was required to achieve marginal improvement, which can be due to the combination of task difficulty and participant fatigue within a trial.

To assess the presence of learning effects across repeated trials, we used permutation tests with ${10}^{6}$ repetitions on the slopes of lines fitted to response time vs. trial number. Regression line slopes for data in the order of performed trials (observed slopes) were compared against distributions of slopes of lines fitted to permuted data, in which response times were randomly reassigned to trial numbers. The permutation tests were applied to successful trials with each mode and subject. Successful trials in experiment 1 are those in which the target was found; in experiment 2 those in which the three obstacles were found and avoided. If the permutation test does not detect a difference between the observed slope and the distribution of randomized order slopes (*p* > 0.05), no learning effect or sign of fatigue over repeated trials was demonstrable through this test. It should be noted, however, that failure to detect such an effect does not prove the absence of the effect.

The data analysis was done in MATLAB version R2022b.

### Ethical approval and consent to participate

The study was approved by the Johns Hopkins Medical Institutions IRB and followed the tenets of the Declaration of Helsinki. All participants signed an informed consent form to participate after being informed of the goals and methods of the study.

## Results

Three participants completed all trials in both experiments. Subjects S1, S2, and S4 completed Exp 1 with a thermal camera and unfiltered video. S3 completed Exp 1 with the thermal camera but only half of the trials with the unfiltered video, due to lack of time. S1 did not participate in Exp 2 due to difficulty walking at the time of testing, while the other three subjects completed this task with the distance filter and unfiltered video.

**Figure 4:**
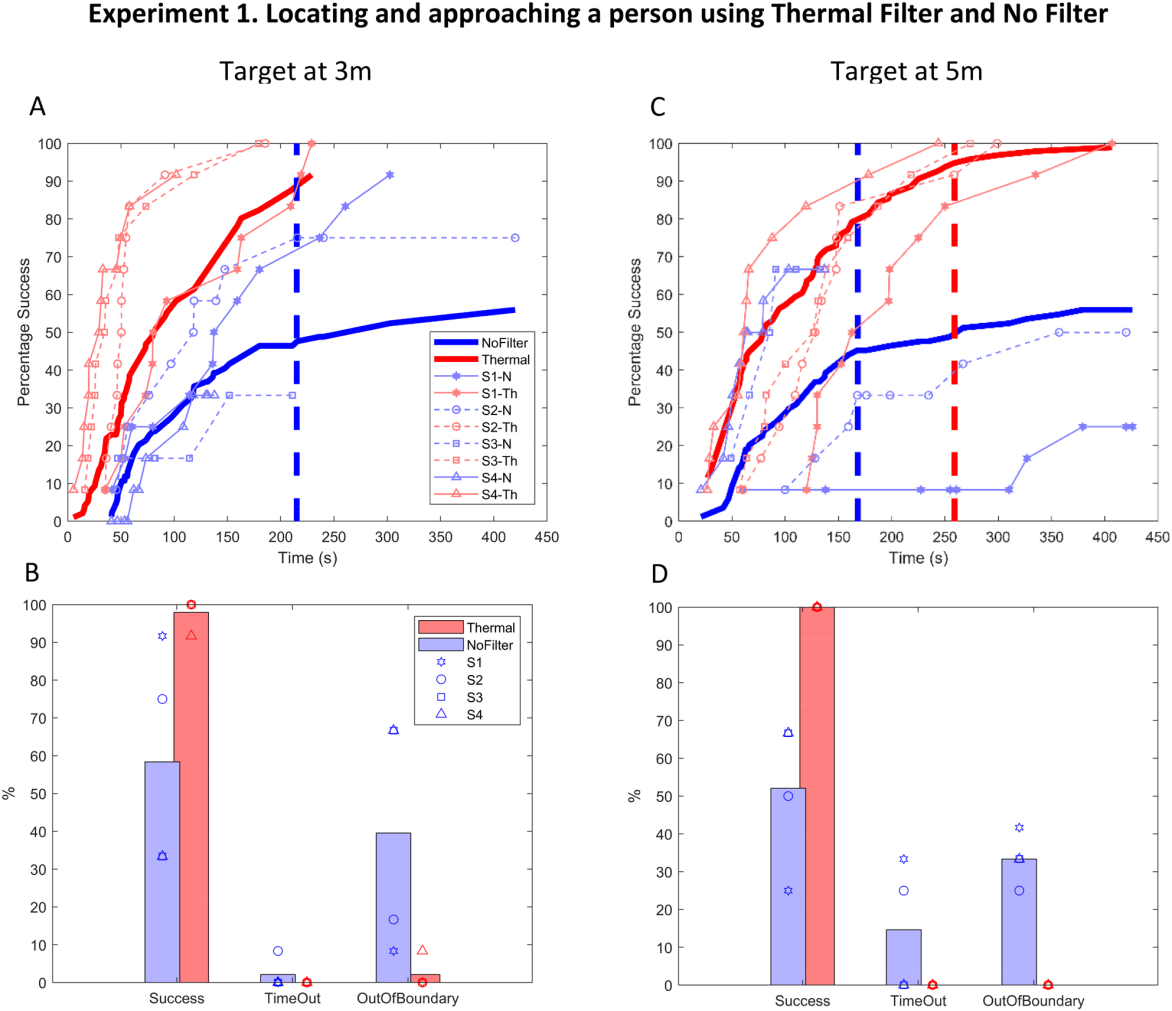
(**A** and **C**) Percentage success versus time for the first experiment for 3 m and 5 m target distances, respectively. The bold lines represent the average performance, the bold dashed lines represent the time when the cumulative probability of success curve reached a plateau. The thin lines represent the individual participants’ performance. Performance with the thermal camera is shown in red, with unfiltered video in blue. (**B** and **D**) Bar graphs comparing the percent correct, time out, and out of boundary trials with the thermal camera in red and unfiltered video in blue for 3 m (**B**) and 5 m (**D**) target distances. Data from individual participants’ performance are indicated by the symbols shown in the legend.

Experiment 1: Thermal filtering vs. unfiltered video—Participants successfully located and approached the target person in 99% of the trials using the thermal camera. Only S4 went outside the testing boundary in one trial with the thermal camera (due to fast walking speed). With unfiltered video, 55% of the trials were completed successfully: S1 and S2 did not find the target within 7min in 17% of the trials, and all subjects went out of the boundaries in 36% of the trials on average. Figure 4a and c show the cumulative probability of success curves when the target was at 3 m and 5 m, respectively. The Kolmogorov–Smirnov test confirmed the significant differences between the performance with the thermal camera and unfiltered video (Table 2), except for S1 when the target was at 3m. As the thin lines in Fig. 4a and c indicate, there are differences between subjects’ performances. However, the thermal camera improved the performance of all individuals regardless of the individual ability to perform the task. Figure 4b and d compare completed and unsuccessful (due to either time-out or walking outside the boundary) trial percentages for thermal filtering vs. unfiltered video, as well as individual performance levels separated according to the target distance.

**Table 2:** Kolmogorov–Smirnov results for unfiltered video vs thermal filtering condition.

	Target at 5 m		Target at 3 m	
Subject	Kolmogorov–Smirnov statistic	P_Value	Kolmogorov–Smirnov statistic	P_Value
All Subjects	D(44,42) = 0.74	5.02E-15	D(44,24) = 0.78	9.77E-09
S1	D(44,42) = 0.97	4.70E-07	D(32,24) = 0.13	0.979
S2	D(44,31) = 0.71	0.0133	D(44,20) = 0.88	1.14E-08
S3	D(15,29) = 0.57	1.33E-04	D(23,19) = 0.94	6.92E-08
S4	D(15,26) = 0.74	1.18E-10	D(15,12) = 0.91	9.30E-05

When the target was at 3 m while using a thermal camera, no plateau was detected on the cumulative probability correct curve for individuals and across subjects; with unfiltered video, the plateau was detected for S4 at (118 s, 3%) and across subjects at (215 s, 48%). When the target was at 5 m while using a thermal camera, the plateau was detected only for the average across subjects at (259 s, 95%); with unfiltered video, the plateau was detected for S1: (379 s, 25%), S3: (91 s, 67%) and across subjects at (168 s, 45%). The plateau wasn’t detected for other subjects.

In experiment 1, no learning effect or sign of fatigue in the response time of finding the target person was found while using the unfiltered video or thermal camera for four participants using a permutation test (Table 3).

**Table 3:** Permutation test results for experiment 1.

	No filter		Thermal filter	
	Target at 3 m	Target at 5 m	Target at 3 m	Target at 5 m
Subject	P_Value	P_Value	P_Value	P_Value
S1	0.35	0.55	0.14	0.21
S2	0.81	0.96	0.99	0.99
S3	0.10	0.90	0.47	0.07
S4	0.30	0.93	0.06	0.35

**Figure 5:**
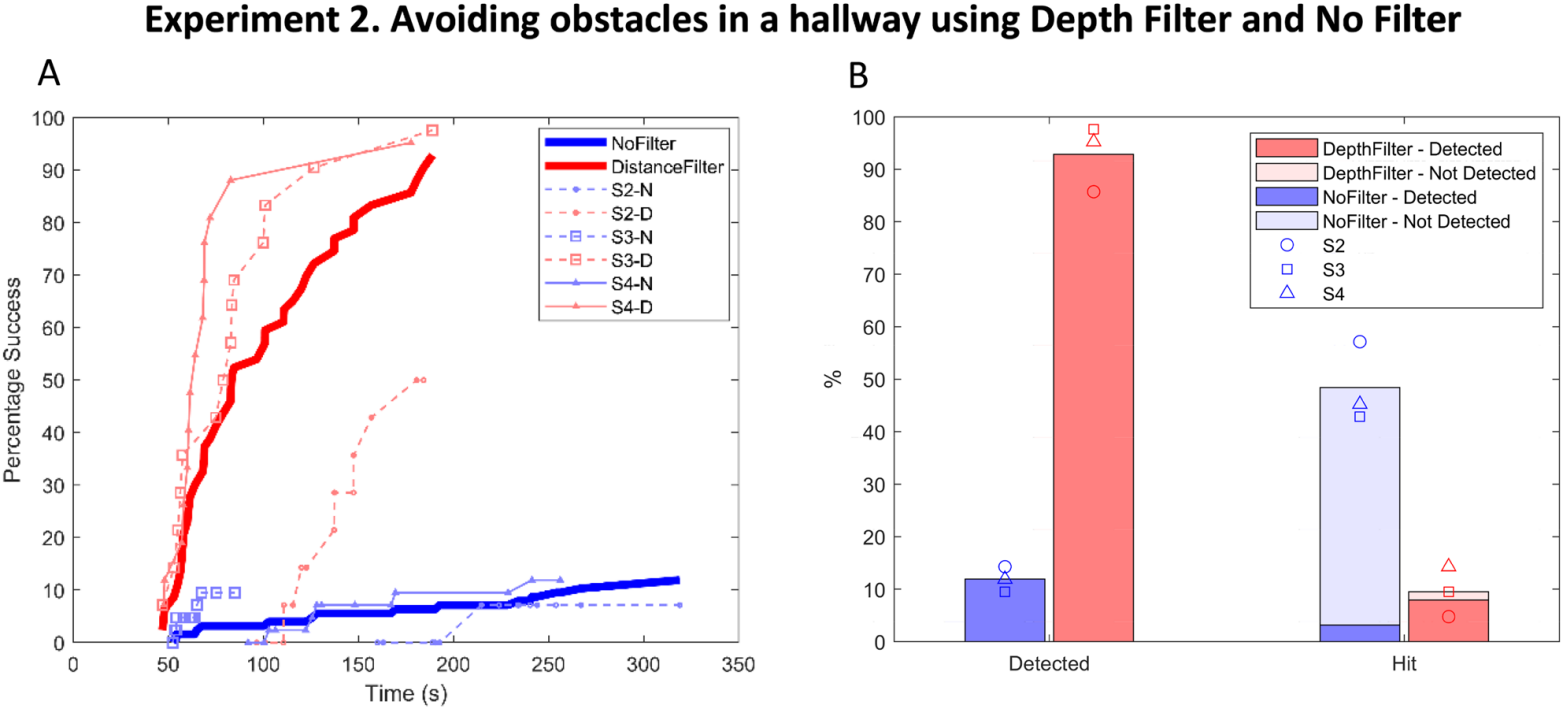
(**A**) The percentage success versus time for the obstacle avoidance experiment. Bold lines represent the average performance across participants; thin lines represent individual participants’ performance. (**B**) Performance with the distance filter is shown in red and with unfiltered video in blue. The bar graph shows the percentage of the obstacles that were detected and the percentage of the obstacles that were hit while it was detected (rich solid color) or not-detected (lighter faded color).

Experiment 2: Distance filtering vs. unfiltered video—The cumulative probability of success over time shows significant differences between the obstacle avoidance performance (which was to detect and avoid three obstacles within a trial) while using the distance filter and while using unfiltered video, both within and across subjects using the Kolmogorov–Smirnov test ($p<0.01$; Table 4 and Fig. 5a). Even though there are interindividual performance differences (as shown in Fig. 5a in thin lines), the distance filter improved the performance of all individuals compared to when they used unfiltered video. Participants successfully detected and avoided 88% of obstacles with the distance filter and 10% of obstacles with unfiltered video. Participants detected 93% of obstacles with the distance filter and 12% with unfiltered video. With the distance filter, 9.5% of obstacles were hit, 1.6% without prior detection, 7.9% despite detection. With unfiltered video, 48% were hit, 45.2% without prior detection, 3.2% despite detection (Fig. 5b).

**Table 4:** Kolmogorov–Smirnov results for unfiltered video vs distance filtering conditions.

Subject	Kolmogorov–Smirnov statistic	P_Value
All subjects	D(33,20) = 0.93	1.80E-08
S1	NA	NA
S2	D(33,20) = 0.80	2.13E-04
S3	D(10,20) = 0.93	2.50E-03
S4	D(27,19) = 0.793	7.40E-07

The cumulative probability of the correct curve didn’t asymptote for the average subject with both conditions, and no plateau was detected for individual subjects when using the distance filter. The plateau was detected with unfiltered video for S2 (214 s, 7%) and S3 (67 s, 10%).

In experiment 2, using the permutation test, no learning effects or signs of fatigue were found with unfiltered video or distance filtering for all participants ($p>0.05$; Table 5).

**Table 5:** Permutation test results for experiment 2.

	No filter	Distance filter
Subject	P_Value	P_Value
S1	NA	NA
S2	1.00	0.17
S3	1.00	0.22
S4	0.60	0.69

## Discussion and conclusions

With a thermal camera, only warm objects will be visible as a bright flash of light in a dark image background, regardless of the ambient lighting and contrast in the room^18^. Therefore, it helps to detect warm objects in the room, e.g., a hot meal, a hot cup of coffee, or people. There are two recently published studies that show the benefit of a thermal camera in stationary tasks^19,20^. The results of the study by He et al., 2020 also suggested that a heat-sensitive camera can help avoid or approach people over distances up to 2.7m. He et al. did not examine, however, the effects on time required for task completion or performance at greater distances without the aid of a cane, which we tested here. We investigated a possible benefit of using a heat-sensitive camera for an O&M application: Can the user locate and approach a person using only visual cues?

The disparity-based distance filtering system detects objects at different distances. It can selectively show those within an adjustable range, making it easier to detect these objects than with unfiltered video. A study by Kartha et al., 2020^21^ reported the benefit of the distance filter camera in stationary tasks; for example, it showed that a distance filter helps with an orientation task to find an object among different-sized objects placed at 1 m; here, we expanded beyond simple orientation and explored a distance filter’s benefit in an obstacle avoidance task while walking. We compared the performance between filtered and unfiltered conditions by varying trials so the participants wouldn’t have prior knowledge before starting a test trial. In real life, detecting an obstacle, even without knowing what objects are, helps to orient oneself in a room and walk confidently, avoiding injuries caused by hitting the objects.

We combined the thermal- and distance-filtering systems along with unfiltered video, similar to that of the camera used in the Argus II system, in one mobile device. Each of the added filter modes has its applications and is useful in different scenarios, thus complementing the unfiltered video provided by the normal Argus II camera. In our tests, the experimenter switched the mode among unfiltered, thermal-, and distance-filtered video, but a future production version of the system will provide a sound notification allowing the user to switch among modes. The other possibility is to overlap the distance filter and thermal filter to the unfiltered video. However, this would require users to perceive multiple gray scales to distinguish the perception with different modes. To date, no study has examined how well participants with Argus II implant can distinguish different gray levels. In this study, we used two levels, black and white, to present the filtered videos.

Since there are few people with Argus II implant in United States, only two local and two out of state people with Argus II were available to participate in this study; for each experiment three participants were able to complete all the trials with both unfiltered and filtered video. All four are highly skilled Argus II users that have participated in many other Argus II related studies prior to these experiments and have been using their system frequently for more than 5 years. Functional vision improvement with filtered video for Argus II users may depend on age, residual light perception, familiarity with the Argus II system, and whether they are using a white cane. We couldn’t study the effect of these factors due to the limited number of participants. We observed improvement for all participants using distance and thermal filters compared to unfiltered video.

Before each task, the participants practiced four and three trials in experiments 1 and 2, respectively. The tasks were designed to perform as many trials within a session as feasible (24 trials for finding a person and 14 trials for the obstacle avoidance task with each mode). There were breaks in between trials to avoid fatigue, and response time data from participants did not show learning effects. The cumulative success rate over the time curve with unfiltered video reached a plateau sooner than with a thermal camera if the plateau was detected. This suggests that the tasks were more difficult with no filter compared to filtered video. Our results demonstrate that participants completed the tasks with higher success rates within any given time using a filtered video.

The design of both tasks aimed to assess the benefits of filtered compared to unfiltered video in settings similar to real-life situations, and they were performed within a hospital hallway. The tasks were intentionally done in less-controlled settings, to replicate the challenges encountered in everyday experiences. The potential confounders in the two tested scenarios included but were not limited to: auditory cues from the target person moving, standing, and breathing through (partially quite long) trials in experiment 1 or while placing objects in experiment 2; other auditory cues while waiting for the next trial; the echo of footsteps; and the space limitations created by the walls. Despite the inherent limitations of less-controlled tasks, we implemented measures to mitigate confounding variables, created each trial as a unique instance, and systematically repeated the procedures under the two conditions, with and without filtered video. Auditory information, for example, would have been equally available in filtered and unfiltered conditions and would have reduced the dramatic difference in performance. While alternative approaches, such as virtual reality, were considered, we opted for a real-world setting to capture the nuances of the real-life scenarios; this also avoided the complexity of integrating the cameras and Argus II VPU into the VR hardware and software environment.

One of the limitations of this study is that the thermal camera was used to find a person standing at 3 or 5 m away in an indoor setting. In real-world scenarios, a user might need to detect a person at greater distances in various indoor and outdoor settings, and in the presence of more clutter. The maximum distance at which the system can detect a warm target depends on its surface temperature. Therefore, the system might detect a person in summer with light-weight clothes but won’t reliably detect a person with winter clothes. This would also limit use of the system in a hot day outdoor setting where the pavement and buildings will be hot enough to be detected and shown as flashes of light, or in a cold winter, when it won’t detect anything. In the current system, the detectable temperature range is adjustable and can be set by the user based on the temperature of the target object, but this needs to be tested in different environments.

Similarly, the distance filter was used to detect a printed random black and white pattern, whereas in daily life objects will be found with different textures. The distance filter operates based on two visible-light sensitive cameras, and distance estimation will be affected by the brightness and detectable detail on the surface of objects. Therefore, it will be important for the system to be tested in various real-life scenarios. Moreover, each person has a different lifestyle and different needs for using the thermal and distance filter system in their routine. It will be important to provide a mobile device that implantees can use in their home environment and while traveling. Users could then provide feedback based on their unique needs to improve the system.

Another limitation of the current system is that it does not include the option of object recognition. Visual percepts from different objects might look the same. So, in real-life scenarios that require finding a specific object, a thermal or distance filter may not help in recognizing the object. As a future direction, with the development of AI technology, we can add an object finder system to identify and notify the specific location of the selected objects.

At the end of each test, we asked the participants if they had any comments about the test. All participants mentioned liking the new modes and expressed an interest in exploring such a system in their home environment when it becomes available. While these initial impressions are promising, a more formal analysis of the new modes' efficacy and potential unmet needs should be performed through a systematic questionnaire once participants utilize the upgraded system in their real-life activities.

Our study showed that participants, when utilizing the thermal camera and the distance filter, demonstrated a higher accuracy in finding a person and avoiding obstacles within a shorter timeframe than with unfiltered video. These findings suggest the advantage of employing thermal and distance filters to enhance performance in orientation and mobility tasks for visual prosthesis users, even for those who do not use other mobility aids.

## Data Availability

All data presented in this study are available through a reasonable request to the corresponding author.
